# Estimating Postoperative Lung Function Using Three-Dimensional Segmental HRCT-Reconstruction: A Retrospective Pilot Study on Right Upper Lobe Resections

**DOI:** 10.3390/jpm15080364

**Published:** 2025-08-08

**Authors:** Aljaz Hojski, Brigitta Gahl, Michael Tamm, Didier Lardinois

**Affiliations:** 1 Department of Thoracic Surgery, University Hospital Basel, Spitalstrasse 21, 4001 Basel, Switzerland; aljaz.hojski@usb.ch; 2 Surgical Outcome Research Center, University Hospital Basel, Spitalstrasse 12, 4031 Basel, Switzerland; brigitta.gahl@usb.ch; 3 University of Basel, 4003 Basel, Switzerland

**Keywords:** 3D lung reconstruction, postoperative pulmonary function prediction, anatomical lung resection

## Abstract

**Background/Objectives:** Can three-dimensional (3D) reconstruction software that simulates postoperative lung volumes more effectively identify suitable candidates for anatomical lung resection compared to conventional methods, particularly in personalized surgical planning? **Patients/Methods:** This single-center pilot study included 20 patients (10 females; age 68 ± 10 years) who underwent segmental or lobar VATS resection of the right upper lobe for NSCLC. Three-dimensional simulations from preoperative HRCT scans were correlated with measured pulmonary function and compared with predictions from the “5% per segment rule” and the model proposed by Brunelli et al. **Results:** Patients (8/20) with increased postoperative FEV1 [2.40 (0.56) vs. 2.30 (0.55)] had a lower proportion of healthy tissue [76 (18)% vs. 89 (10)%, *p* = 0.045] in 3D simulations than those with decreased FEV1. Mean postoperative FEV1 was 2.3 (0.54); the Brunelli model predicted 1.8 (0.36) and the “5% rule” 2.2 (0.54). Both models underestimated postoperative function, though the “5% rule” was more accurate. **Conclusions:** This pilot study suggests that 3D-HRCT reconstruction has the potential to facilitate patient-tailored identification of individuals who may derive greater benefit from surgical intervention compared to conventional methods. Further research is needed to determine whether this technology can more accurately predict postoperative pulmonary function in patients with severe COPD. Utilizing 3D-segmental-HRCT-reconstruction software, the advantages of lung resection in the context of NSCLC can be assessed on an individualized patient basis.

## 1. Introduction

Screening programs, as well as wider and better access to HRCT diagnostics, have led to an increased detection of relatively small lung lesions. A recent randomized controlled trial (RCT) demonstrated that segmentectomy should be considered the treatment of choice for early-stage non-small-cell lung cancer (NSCLC) [1]. Minimally invasive anatomical segmentectomy of the lung spares the lung parenchyma. However, it remains unclear whether this translates into clinically meaningful preservation of pulmonary function on an individual basis. Data gathered during follow-up in this study demonstrated that, albeit to a lesser extent than anticipated (3.5% vs. expected 10%), minimally invasive sub-lobar anatomical lung resection (segmentectomy) preserved lung function better than lobectomy [1].

Segmentectomy is technically more challenging than lobectomy [2] and raises new questions about how the extent of functional parenchyma removal and the subsequent anatomical changes affect the function of the remaining lobes [3,4]. From a personalized medicine perspective, our goal is to determine the necessary parameters to improve the selection of patients who will benefit from either segmentectomy or lobectomy. To avoid unnecessary complications, possible postoperative complications should be considered in the decision-making process [5]. A recent literature review has comprehensively examined the available methods for predicting such postoperative complications following major lung resection [6]. The increasing complexity of surgical techniques must be accompanied by improved prognostic tools to achieve optimal outcomes. While traditional spirometry criteria and perfusion scans (SPECT/CT) remain valuable, they often lack sufficient spatial resolution to capture segmental variability [7]. Another study demonstrated that SPECT/CT can accurately predict postoperative lung function, particularly in patients with limited pulmonary reserve undergoing resection for lung cancer [8]. The incorporation of novel imaging techniques into routine surgical planning could improve prognostic accuracy and enable a transition to function-preserving interventions tailored to each patient’s anatomy and physiology [2]. Software-based CT assessments of well-aerated lung have shown promising results in quantifying predicted pulmonary function in resected NSCLC [9].

The guidelines for surgical planning are based on research data primarily collected from open lung operations and lobar resections. Additionally, patient categorization into low- and high-risk groups is determined by the number of segments to be removed [5]. Current predictive models for postoperative pulmonary function rely on the amount of lung parenchyma to be removed [10,11,12,13,14] and the anatomical changes it induces [3,4,15,16]. Although the loss of functional lung volume per segment can range from 0 to 10%, we occasionally observed improvement in pulmonary function [4]. The degree of variation in postoperative pulmonary function depends on numerous factors [4,5,15,16,17,18], including the following:

- Surgical approach and extent of resection:
  ◦ Type of approach (open vs. minimally invasive)
  ◦ Number of segments removed (ranging from (sub)segmentectomy to entire lobectomy)
  ◦ Localization of the segment(s) to be removed
- Technical aspects of the procedure:
  ◦ Division of the intersegmental vein
  ◦ Vascular supply to the remaining parenchyma
  ◦ Type of parenchymal division (stapler, energy device, electrocautery)
- Patient-specific and functional factors:
  ◦ Presence and distribution of parenchymal disease
  ◦ Compensatory lung response, including:
    - Cooperation of existing alveolar-capillary reserves
    - Remodeling of the remaining airways
    - Increase in the function of the remaining lung tissue

The right upper lobe is the most extensively studied [3] and is the most likely to be susceptible to the consequences of lung volume reduction [17]. In this context, the emergence of advanced 3D visualization and modeling tools such as Visible Patient (VP) software represents a significant step forward in personalized preoperative planning. VP uses high-resolution computed tomography (HRCT) with contrast enhancement data to generate accurate, patient-specific anatomical reconstructions. This enables precise visualization of bronchovascular structures at the segmental level and allows the surgeon to interactively define and adjust the extent of the planned resection, tailoring it to the patient’s unique anatomy and tumor location. One of the key features of the software is its ability to compute volumetric data based on Hounsfield unit thresholds, allowing for functional mapping of lung parenchyma in the range of −900 to −501 HU. Voxels outside this range were considered either emphysematous (<−900 HU) or poorly aerated/non-aerated (>−500 HU). This quantitative assessment not only aids in surgical planning but also facilitates simulation of different resection scenarios with estimated impacts on postoperative lung function.

Postoperative FEV1 and DLCO values can be estimated either using the traditional “5% rule,” which assumes that each lung segment contributes approximately 5% to total lung function, or using more complex regression models, such as those developed by Brunelli et al. [5,19]. In their multivariate model, the estimated percentage FEV1 reduction is calculated using the following formula:

FEV1 loss (%) = 21.34 − (0.47 × age) + (0.49 × % of removed functioning lung parenchyma) + (17.91 × COPD index),

While the estimated DLCO reduction is calculated as follows:

DLCO loss (%) = 35.99 − (0.31 × age) − (36.47 × FEV1/FVC) + (0.33 × DLCO) + (0.54 × % of removed functioning lung parenchyma).

These models highlight the multifactorial nature of postoperative lung decline and account for patient-specific physiological variables as well as the extent of resection. However, a key prerequisite for the precise application of these formulas is a precise estimation of the percentage of functional lung parenchyma removed—a task often performed only approximately in the clinical setting. This is precisely where individualized, high-resolution 3D HRCT segment reconstruction offers a decisive advantage. By quantifying the functional contribution of the lung segments under investigation in detail, our method improves the accuracy of the input variables (i.e., the percentage of functional lung parenchyma removed), thus increasing the predictive accuracy of such models.

The aim of this pilot study was to estimate postoperative lung volumes based on preoperative assessment of the lung parenchyma using individualized 3D-HRCT reconstruction and compare these with estimations of the conventional methods and actual postoperative measurements. Conventional methods include standard spirometry with body plethysmography (to assess DLCO and RV), HRCT and PET-CT imaging for staging and anatomical assessment, perfusion scintigraphy (SPECT-CT when indicated), and 3D reconstructions for anatomical surgical planning [5,20].

## 2. Materials and Methods

### 2.1. Study Subjects

Following approval from the local ethics review board EKNZ (Ethikkommission Nordwest- und Zentralschweiz, BASEC 2022-01743, granted on 19 October 2022), 20 patients who signed the general research consent form of the University Hospital Basel and underwent a VATS anatomic lung resection of the right upper lobe between 29 October 2020 and 3 October 2022, at the University Hospital Basel, were included. The patient cohort consisted of individuals undergoing resection primarily for early-stage NSCLC. Demographic data (age, gender, BMI), type of surgery performed (lobectomy, segmentectomy—including the number of segments removed) and pulmonary function were recorded to assess baseline comparability and variability. The inclusion criteria required that patients had complete preoperative and six-month postoperative pulmonary function tests available, high-resolution (HR)CT imaging suitable for 3D reconstruction, and no additional pulmonary surgical procedures during the follow-up period. Patients with preexisting interstitial lung disease, prior lobectomy, or segmentectomy were excluded from the analysis.

### 2.2. Study Design

This single-center retrospective study serves as a feasibility analysis to evaluate the diagnostic value of 3D-HRCT-segmental reconstruction in patients undergoing minimally invasive surgery for NSCLC. No formal sample size calculation was performed, as the primary aim was to generate preliminary data to inform future adequately powered prospective studies. The study was not powered for outcome comparisons but designed to explore whether volumetric estimates from HRCT-based segmental reconstruction correlate with functional outcomes. The core hypothesis was that a detailed 3D reconstruction of the lung anatomy and parenchymal function could enhance individualized surgical planning and improve the prediction of postoperative lung function. For preoperative assessment, conventional methods included standard pulmonary function tests (spirometry and body plethysmography to assess FEV1, DLCO, and RV), as well as imaging with HRCT and PET-CT for anatomical and oncological staging. Perfusion scintigraphy (SPECT-CT) was performed when clinically indicated. These conventional assessments were used as a reference for comparison with the 3D-HRCT-based volumetric reconstruction performed in this study. Findings from this pilot cohort are intended to design of a prospective study evaluating individualized preoperative planning for anatomical lung resections.

### 2.3. Methods

Clinical data were retrieved from the electronic medical records (operative reports, pulmonary function test reports, etc.) of the University Hospital of Basel. Specific data points extracted included the extent of resection (removed segments), intraoperative findings, and any perioperative complications. This provided a comprehensive clinical context for the interpretation of functional outcomes. Standard contrast-enhanced (venous or arterial phase) HRCT scans with a thickness of 1–1.5 mm were anonymously exported from the PACS system. These scans were acquired during full inspiration using a standardized protocol to ensure reproducibility across all patients. After pseudo-anonymization, the HRCT scans were converted into 3D lung reconstructions using Visible Patient software (VP-Planning™ software, certificate N°28229 rev 8, a certified Class II.A medical device, Strasbourg, France). Removed and remaining functional lung parenchyma volume was determined retrospectively based on the type of surgery performed. A single experienced thoracic surgeon performed segmentation and volumetric analysis of both resected and preserved functional lung parenchyma using the 3D reconstructions. This approach ensured consistency in interpreting segmental anatomy and minimized inter-operator variability, particularly in delineating intersegmental borders.

The HU-based approach distinguishes between well-ventilated, poorly ventilated, and non-ventilated lung parenchyma, thus providing an indirect measure of segmental functional reserve. This method has been previously validated in various clinical contexts, for example, in patients with COVID-19-related ground-glass opacities (14), and was adapted here to estimate residual functional parenchyma and the functional loss after resection.

Preoperative and 6-month postoperative pulmonary function was measured via body plethysmography (Jaeger^®^ Master Screen Body, Mannheim, Germany). All pulmonary function tests were performed at the Department of Pulmonology, University Hospital Basel, by trained respiratory technicians. The interpretation of the spirometry measurements was conducted according to the GLI-2017 (Global Lung Initiative of the European Respiratory Society) equations [21].

### 2.4. Analysis

We summarized continuous variables as means with standard deviations, and categorical variables as numbers with percentages. There was no missing data for the 20 patients included in the study. To compare differences between patients with higher versus lower postoperative FEV1 compared to preoperative values, we used an unpaired Student’s T-test. Statistical significance was defined as a *p*-value < 0.05. We plotted the data as a scatter plot with a line of best fit and a line of no change. Stata 16.0 (StataCorp, 4905 Lakeway Drive, College Station, Texas) was used as statistical software.

## 3. Results

**Figure 1:**
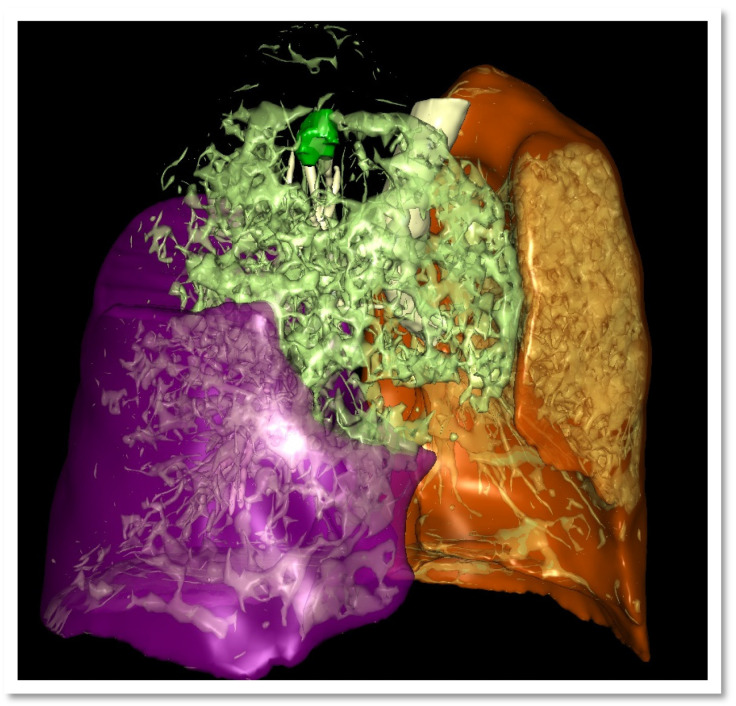
A case of preoperative reconstruction and simulation of right upper lobectomy using VP software with additional tissue quality identification. Legend: brown—left lung, violet—right lower and middle lobe, dark green—tumor, light green—poorly-functional parenchyma right upper lobe, white—airway.

Pre- and postoperative data were collected from 20 consecutive patients who underwent minimally invasive surgery of the right upper lung lobe between October 2020 and October 2022. The mean (SD) age was 68 years (10), with an equal distribution of females and males (50% each). Fourteen patients (70%) underwent a right upper lobectomy, while six (30%) received a segmentectomy, including two bi-segmentectomies (Table 1). Figure 1 provides a representative 3D reconstruction example using the VP software, including tumor localization and functional tissue mapping. All operations were performed for NSCLC. The average number of segments resected was three in 70% of cases, two in 10%, and one in 20%.

**Figure 2:**
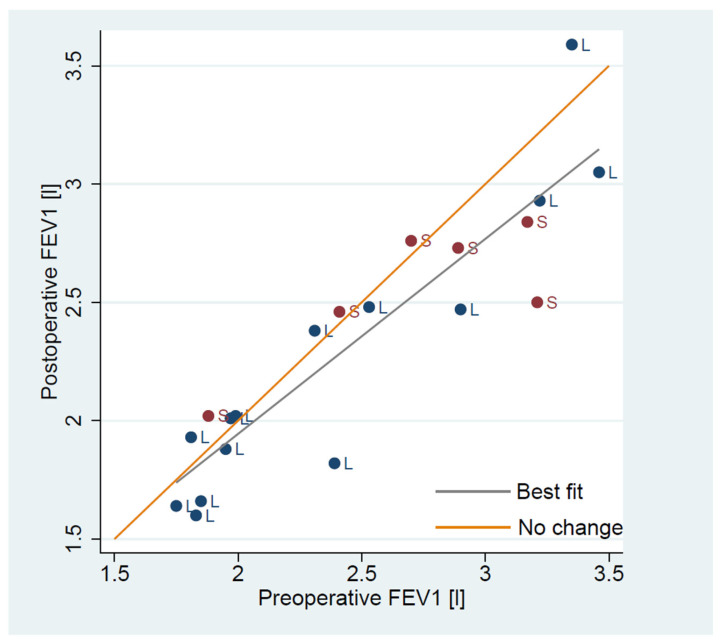
Preoperative to postoperative measured FEV1 [l] change. Legend: segmentectomy—S, lobectomy—L.

**Figure 3:**
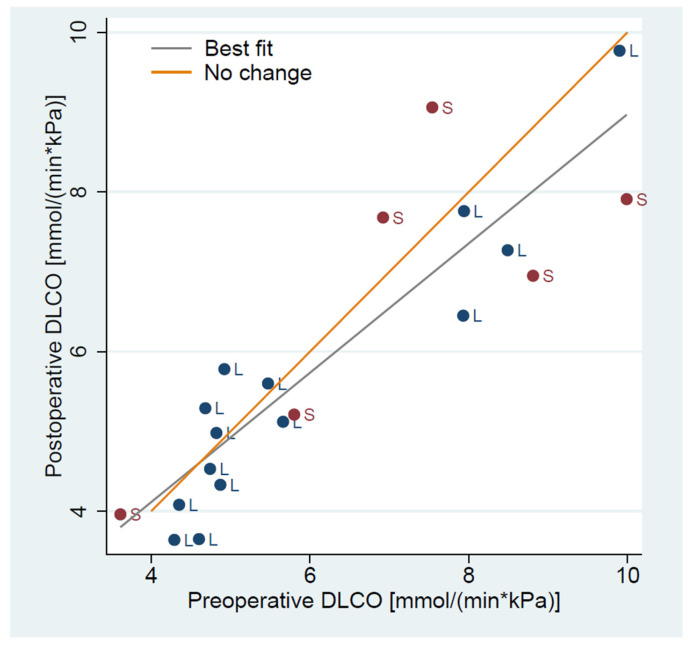
Preoperative to postoperative measured DLCO [mmol/(min∗kPa)] change. Legend: segmentectomy—S, lobectomy—L.

Preoperative pulmonary function tests revealed a mean FEV1 of 2.48 L (0.59), corresponding to 92% (15%) of predicted. The mean DLCO was 6.27 mmol/min·kPa (1.99), or 81% (22%) predicted. The FEV1/FVC ratio averaged 0.69 (0.09), and the mean COPD-index, as defined by Korst et al. [22], was 1.61 (0.21). Based on 3D reconstruction data, the average simulated resection comprised 17.21% (6.05%) of total lung tissue and 16.42% (6.36%) of the functional parenchyma. The mean postoperative FEV1 measured at 6 months was 2.34 L (0.54), or 89% (17%) predicted, reflecting a mean loss of only 5% (9%) relative to baseline. In contrast, the Brunelli model predicted a 26.4% decline in FEV1, equivalent to 1.8 L (0.36), while the simplified “5% rule” estimated 2.2 L (0.54). Hence, both methods underestimated the postoperative volume, though the “5% rule” was more closely aligned with the measured value than the Brunelli model. DLCO at 6 months post-surgery remained relatively stable, with a mean of 5.95 mmol/min·kPa (1.82), or 79% (21%) predicted (Table 1). Figure 2 shows individual changes in FEV1 by patient and operation type, while Figure 3 illustrates changes in DLCO.

Among the 20 patients, we identified eight patients (8/20) whose postoperative pulmonary function measurement showed an increase in FEV1 compared to their preoperative baseline. These were analyzed separately from the 12 patients with a postoperative decrease in FEV1 (Table 2). Further assessment of the eight patients with improved postoperative FEV1 showed that the majority had some degree of pre-existing obstructive ventilatory impairment and elevated residual volumes, which are suggestive of hyperinflation. In these patients, the resected lung tissue predominantly consisted of non-functional or poorly ventilated parenchyma, as indicated by their preoperative 3D reconstructions. In the FEV1-increase group, mean FEV1 rose from 2.30 L (0.52) to 2.40 L (0.56), while in the decrease group, it dropped from 2.60 L (0.63) to 2.30 L (0.55). Notably, preoperative VP simulation showed that the increase group had a significantly lower proportion of functional tissue removed (76% [18]) than the decrease group (89% [10], *p* = 0.045). Other simulated volume parameters, such as ipsilateral residual functional lung, followed similar trends (Table 2).

Postoperative FEV1 calculations [now in ml difference] using the “5% rule” underestimated the measured values by 360 mL in the increase group, whereas in the decrease group, the measured and calculated values differed only by 40 mL. The Brunelli model showed an even greater deviation, underestimating postoperative FEV1 by 660 mL in the increase group and 450 mL in the decrease group, respectively (Table 2). In the increase group, residual volume (RV) decreased by 480 mL from 128% predicted to 107%, whereas in the decrease group, RV dropped by only 200 mL (113% to 104% predicted). However, this difference was not statistically significant (Table 2).

The mean postoperative DLCO [mmol/min∗kPa] remained stable in the FEV1-increase group at 6.34 (2.28) from 6.32 (2.51), whereas in the FEV1-decrease group, it declined to 5.69 (1.49) from 6.23 (1.68) (Figure 3). The mean postoperative DLCO of the FEV1-increase group was comparable to the Brunelli model prediction of 6.32 (2.73), whereas in the FEV1-decrease group, the Brunelli model overestimated the postoperative DLCO, 6.20 (1.69), as compared to 5.69 (1.49). The “5% rule” failed in both groups, estimating DLCO at 5.63 (2.37) and in the FEV1-increase group, and 5.43 (1.58) in the FEV1-decrease group.

## 4. Discussion

The aim of this pilot study was to examine the relationship between preoperative software-based 3D simulations and postoperative functional lung volume using HRCT scans. Preoperative lung function tests remain essential for the assessment of patients undergoing anatomical lung resection [5], as they help predict surgical risk in patients with limited pulmonary function. The algorithm was created based on lobectomies and pneumonectomies via thoracotomy. However, standard surgical approaches have evolved towards minimally invasive procedures and, more recently, sub-lobar resection [1]. As a result, decision-making must be adapted, as postoperative outcomes following minimally invasive sub-lobar resections are not directly comparable to those of open major lung resections. The increasing availability of HRCT in recent years has facilitated the detection of early-stage NSCLC in elderly and multimorbid patients who may benefit from segmental or lobar resection using VATS [20]. Therefore, current guidelines may have to be adapted in the near future [5].

To provide patients with a safe procedure and reduce postoperative complications, we have implemented preoperative 3D imaging for pre- and intraoperative planning [20]. This approach allows for a better understanding of patient-specific anatomy, precise determination of resection margins, and strategic planning of a safer surgical approach [20]. Such individualized planning reflects a core principle of personalized thoracic surgery, which aims to match the extent and method of resection to each patient’s unique anatomical and functional characteristics. With the newly available VP reconstruction software [23], preoperative functional lung volume can be estimated at the (sub)segmental level [18]. This enables better assessment of lung parenchyma loss caused by surgery and may improve various prediction models [5,6,19]. The ability to simulate postoperative outcomes preoperatively supports personalized decision-making, especially in borderline or high-risk patients. This may be particularly valuable in the assessment of patients with severely impaired pulmonary function [22].

Previous work has demonstrated that such automated lung volume analysis can be performed significantly faster than traditional methods while maintaining excellent reliability, with intraclass correlation coefficients exceeding 0.99 [18]. This degree of automation and accuracy is particularly valuable in clinical environments where time and reproducibility are critical. Furthermore, the integration of anatomical and functional data offers an unprecedented opportunity to model the trade-offs between parenchymal preservation and oncologic safety with greater precision. Traditional estimation of postoperative function is typically based on segment counts and assumes equal contribution of each segment to overall lung function. However, this assumption does not hold in patients with regional emphysema, fibrosis, or infection-related changes. The 3D-HRCT tool enables volumetric quantification that reflects true functional contribution and anatomical variability, potentially improving prediction accuracy and enabling more individualized surgical planning.

The right upper lobe was selected as the focus of this study due to its relatively standardized and well-described segmental anatomy, which facilitates consistent imaging-based reconstruction and surgical planning. Additionally, previous studies have highlighted this lobe’s disproportionate influence on postoperative pulmonary function, making it a clinically relevant model for evaluating functional outcomes following anatomical resection [14,15,16,17].

Our findings indicate that conventional models fail to accurately predict postoperative pulmonary function. The Brunelli calculation, despite its complexity, was not reliable in our patient group [19]. Similarly, while the “5% rule” is simple, it is not universally applicable, especially in patients with significant emphysema [3,4,5,15,16,17]. This trend highlights the impact of functional rather than anatomical volume in predicting postoperative performance.

In our analysis, a significant proportion of patients (8/20) experienced improved pulmonary function following surgery. Further evaluation of these patients showed that their preoperative scans demonstrated significant destruction or hyperinflation of the resected segments. These results imply that the removal of hyperinflated, non-functional lung segments may have improved overall pulmonary mechanics by reducing air trapping and enhancing ventilation efficiency. However, due to the small sample size, these findings should be interpreted cautiously and require confirmation in larger cohorts. This paradoxical improvement underscores the limitations of general prediction models and highlights the need for more patient-tailored assessments. Current predictive models may lead to the exclusion of patients with severe COPD and NSCLC from curative surgery, despite the potential for postoperative improvement in their pulmonary function. While not statistically significant, this trend supports the hypothesis that lung volume reduction due to loss of overinflated, nonfunctional areas may contribute to functional gains. Our patients who demonstrated improved postoperative pulmonary function also showed a substantial reduction in RV. This aligns with the fact that their 3D reconstructions showed more destroyed lung tissue, subsequently resected during surgery. Additionally, this group of patients exhibited no deterioration in postoperative diffusion capacity.

The right upper lobe is easily accessible for segmentectomy, and the effects of volume reduction in this region are well-documented [3,15]. Therefore, our hypothesis was tested under these conditions. However, in the spirit of personalized medicine, it is critical to expand evaluation beyond anatomical accessibility to include individual parenchymal integrity, segmental ventilation, and compensatory potential. Together, these findings support the feasibility and potential added value of individualized 3D volumetric analysis in predicting functional outcomes, particularly by better estimating the proportion of functional lung tissue removed, which appears to explain outcome variation more reliably than anatomical resection volume alone. Building upon these developments, integration of artificial intelligence and machine learning into quantitative imaging and 3D reconstruction workflows could enable automated segmentation, improve predictive accuracy, and optimize surgical planning. Recent analyses have emphasized the role of advanced imaging modalities, such as SPECT/CT and quantitative CT, in improving preoperative evaluation [8,9,11]. Combining these imaging biomarkers with deep learning models could facilitate patient-specific predictions of postoperative lung function and complication risk, as well as adaptive learning based on individual surgeons’ techniques and outcomes. This would create a dual personalization approach that supports both patient selection and improvement in surgical performance. Realizing this vision will require close collaboration with industry partners developing 3D planning software, as well as with research groups specializing in quantitative imaging and deep learning modules. Further research is required to investigate the possible advantages of 3D reconstruction analysis in evaluating postoperative lung function in patients who have undergone lung surgery on lobes other than the right upper lobe. Procedures to reduce lung volume are widely accepted, with proven clinical benefits. Korst et al. have shown that some patients with emphysema experience a lower-than-expected loss of FEV1 following lobectomy when using the “5% rule” for prediction [22]. Nevertheless, current guidelines for selecting patients eligible for curative surgery for NSCLC do not adequately account for the impact of lung volume reduction.

Three-dimensional reconstruction tools provide an opportunity to shift from a generalized, population-based surgical risk assessment toward individualized, data-driven decision-making. This technology may assist in identifying patients with NSCLC who remain operable despite the presence of advanced COPD by providing a comprehensive evaluation of both functional and non-functional lung tissue [18,20]. A key objective of our study was to identify tissue parameters that represent the relationship between functional and non-functional parts of the lung, which could be tested in further studies and, when validated, used in clinical practice. Such parameters could form the basis for integrating radiological-functional modeling into routine thoracic surgical planning, making it more responsive to individual patient profiles.

A limitation of our study was that only a small number of patients with good preoperative pulmonary function who underwent right upper lobe surgery were included. Larger studies are needed to determine whether 3D reconstructions better predict postoperative lung volumes in patients with advanced COPD and reduce postoperative complications. Additionally, further validation of these findings is required in patients undergoing segmentectomy or lobectomy in lobes other than the right upper lobe. Prospective multicenter trials incorporating patient-specific modeling into clinical algorithms could help confirm its role in guiding personalized treatment strategies in thoracic surgery. One consideration that may be worthy of further discussion is the accessibility and cost of specialized 3D reconstruction software. This may have implications for its wider application, particularly in settings with limited resources.” However, in the future, such applications could potentially replace other, more costly or less accessible tests, such as SPECT-CT, for preoperative functional assessment in patients with limited pulmonary reserve.

## 5. Conclusions

This pilot study shows that the 3D reconstruction software VP has the potential to more accurately identify patients who might benefit from surgery compared to traditional methods, specifically in the context of right upper lobe resections. By enabling individualized, segment-level analysis of functional and non-functional lung parenchyma, this approach supports more personalized surgical planning in patients with early-stage NSCLC. A subset of patients in our study experienced improvement in postoperative pulmonary function. This suggests that current predictive models may underestimate the suitability and benefit of surgery, particularly in patients with advanced COPD. These findings highlight the limitations of conventional methods, such as the “5 percent rule” and the advanced models, such as the Brunelli formula, which do not fully account for heterogeneous parenchymal damage or the potential for compensation.

Three-dimensional reconstruction and simulation technology offer a promising tool to support surgical decision-making, particularly in high-risk patients with severe COPD who were previously considered borderline operable. By providing a more sophisticated view of lung function, it can expand access to curative surgery while reducing postoperative complications. However, it is important to note that these findings are currently limited to right upper lobe resections. To confirm the accuracy and clinical utility of the findings, further validation in larger, more diverse patient populations is needed. This would include patients undergoing other lobar resections. Future multicenter prospective studies should evaluate the integration of radiological-functional models into standard clinical algorithms to establish their role in routine thoracic surgery planning and patient selection, minimizing postoperative risk, and expanding access to curative surgical options for high-risk individuals.

## Figures and Tables

**Table 1:** Patient characteristics.

	Total (*n* = 20)
Age, years	68 (10)
Gender *n* (%)	
Female	10 (50)
Male	10 (50)
BMI [kg/m^2^]	27 (4.9)
Type of operation *n* (%)	
Lobectomy	14 (70)
Segmentectomy	6 (30)
Number of segments removed *n* (%)	
3	14 (70)
2	2 (10)
1	4 (20)
Lung function	
Pre-operation	
FEV1 [l]	2.48 (0.59)
FEV1 [%]	92 (15)
DLCO [mmol/(min∗kPa)]	6.27 (1.99)
DLCO [%]	81 (22)
FEV1/FVC	0.69 (0.09)
COPD-index *	1.61 (0.21)
FEV1 loss [%] est. Brunelli	26.38 (6.25)
Removed functioning parenchyma [%]	16.42 (6.36)
Post-operation	
FEV1 [l]	2.34 (0.54)
FEV1 [%]	89 (17)
FEV1 loss [%]	5.0 (9.0)
DLCO [mmol/(min∗kPa)]	5.95 (1.82)
DLCO [%]	79 (21)

* COPD-index calculated according to Korst et al. [22]. Values are given in mean (SD) if not otherwise mentioned.

**Table 2:** Descriptive Statistics of FEV1, DLCO, and simulated organ volume.

		Increase (*n* = 8)	Decrease (*n* = 12)	*p*
FEV1 [l]				
preoperative	measured	2.3 (0.52)	2.6 (0.63)	0.29
postoperative				
	measured	2.4 (0.56)	2.3 (0.55)	0.71
	using 5% rule	2.04 (0.47)	2.26 (0.58)	0.40
	using Brunelli formula	1.74 (0.32)	1.85 (0.40)	0.52
DLCO [mmol/(min∗kPa)]				
preoperative	measured	6.32 (2.51)	6.23 (1.68)	0.93
postoperative				
	measured	6.34 (2.28)	5.69 (1.49)	0.45
	using 5% rule	5.63 (2.37)	5.43 (1.58)	0.82
	using Brunelli formula	6.32 (2.73)	6.2 (1.69)	0.90
RV [l]				
preoperative	measured	2.96 (0.63)	2.60 (0.90)	0.34
postoperative	measured	2.48 (0.58)	2.40 (0.77)	0.80
Preoperative simulated organ volume [%]				
healthy total/total ipsilateral: remaining healthy/remaining total removed healthy/removed total		75 (17)	87 (11)	0.06
		78 (13)	88 (9.1)	0.07
		76 (18)	89 (10)	0.045

Increase and decrease are given in mean (SD). In eight patients, the postoperative FEV1 was higher than the preoperative.

## Data Availability

The data underlying this article will be shared on reasonable request to the corresponding author.

## Abbreviations

The following abbreviations are used in this manuscript:

3D	Three-dimensional
COPD	Chronic obstructive pulmonary disease
DLCO	Diffusion capacity of the lungs for carbon monoxide
FEV1	Forced expiratory volume in 1st second
FVC	Forced vital capacity
HRCT	High-resolution computer tomography
NSCLC	Non-small-cell lung cancer
PACS	Picture archiving and communication system
RCTs	Randomized controlled trials
RV	Residual volume
SPECT-CT	Single photon emission computed tomography, e.g., lung perfusion scintigraphy
VP	Visible Patient^TM^
